# Liposomal and Liposomes-Film Systems as Carriers for Bioactives from *Paeonia tenuifolia* L. Petals: Physicochemical Characterization and Biological Potential

**DOI:** 10.3390/pharmaceutics15122742

**Published:** 2023-12-07

**Authors:** Natalija Čutović, Tatjana Marković, Tamara Carević, Dejan Stojković, Branko Bugarski, Aleksandra A. Jovanović

**Affiliations:** 1 Institute for Medicinal Plants Research “Dr. Josif Pančić”, Tadeuša Košćuška 1, 11000 Belgrade, Serbia; tmarkovic@mocbilja.rs; 2 Department of Plant Physiology, Institute for Biological Research “Siniša Stanković”—National Institute of Republic of Serbia, University of Belgrade, Bulevar Despota Stefana 142, 11000 Belgrade, Serbia; tamara.carevic@ibiss.bg.ac.rs (T.C.); dejanbio@ibiss.bg.ac.rs (D.S.); 3 Faculty of Technology and Metallurgy, University of Belgrade, Karnegijeva 4, 11000 Belgrade, Serbia; branko@tmf.bg.ac.rs; 4 Institute for the Application of Nuclear Energy INEP, Banatska 31b, Zemun, 11080 Belgrade, Serbia; ajovanovic@inep.co.rs

**Keywords:** encapsulation, biological activity, peony, liposomes, biopolymer films

## Abstract

*Paeonia tenuifolia* L. (steppe peony) petal extract was proficiently encapsulated into liposomes and biopolymer films in the current work, both times utilizing a single-step procedure. The encapsulation efficiency, size of the particles, and index of polydispersity (PDI), as well as the ζ potential of the obtained liposomes were determined, whereas in the case of films, the test included moisture content and mechanical property assessment. Fourier transform infrared spectroscopy (FT-IR) was used to evaluate the chemical composition and existence of numerous interactions in the systems. All the obtained encapsulates were subjected to antibacterial, antifungal and antibiofilm activity testing of the pathogens associated with human skin. The results indicated that the liposomes prepared using Phospholipon had the highest encapsulation efficiency (72.04%), making them the most favorable ones in the release study as well. The biological assays also revealed that Phospholipon was the most beneficial phospholipid mixture for the preparation of liposomes, whereas the film containing these liposomes did not have the ability to inhibit pathogen growth, making the double encapsulation of *P. tenuifolia* L. petal extract needless. These findings may be a first step toward the potential use of steppe peony extract-loaded films and liposomes in pharmaceutical and cosmetical industries.

## 1. Introduction

*Paeonia tenuifolia* L., also known as the steppe peony, belongs to the *Paeonia* L. genus of the Paeonicae family [1]. *Paeonia* plants are well known for containing monoterpene glycosides with a “cage-like” pinane skeleton, as well as some other classes of bioactives, such as steroids and polyphenolic compounds [2]. Specifically, the petals of fern leaf peonies proved to be rich in phenolic acids, anthocyanins and anthocyanidins, flavonoids, and terpene derivatives [2,3], which possess antioxidant, antimicrobial, and antibiofilm activities, as well as wound healing potential, and an inhibition effect on the adhesion and invasion of the bacterium *Staphylococcus lugdunensis* [3]. Extracts from the petals of *P. tenuifolia* L. were also proposed as topical treatments for bacterial and fungal-induced skin diseases, to inhibit pathogen adhesion and penetration through the skin surface, and accelerate the healing of wounds [3]. 

In the past, various plant parts of peonies (seeds, flowers, leaves and roots, as well as whole plants) have been widely used to treat various diseases, pain (head, stomach, and eyes) [4], problem regarding female genitals (dysmenorrhea and amenorrhea) [5], aches, neurological diseases (spasm and epilepsy) [6], infectious diseases (carbuncles) [4], urinary system diseases, and inflammation (otitis media, appendicitis, and gastritis) [6], as well as trauma. The petals of herbaceous peonies have been a subject of very few studies, and it has been shown that they possess skin-beneficial biological activities [3,7].

Despite the fact that plant extracts are useful for treating a variety of disorders, research demonstrated that their therapeutic value is restricted due to their complicated composition and toxicity when administered to organisms with more complex metabolic systems. Furthermore, organic solvents (e.g., methanol, ethanol, hexane, dichloromethane, ethyl acetate, etc.) are commonly utilized to generate these extracts. As a result, the final vehicle in which the extracts are contained prohibits them from being directly applied in organisms [8], and their encapsulation is needed. In addition to the limited bioavailability and instability of flavonoids, phenolic acids, and anthocyanins present in *P. tenuifolia* L. petal extracts [9], as well as their limited bioavailability, carriers for the extract ought to be created in order to protect the biologically active components and enable more comfortable dermal applications. To accomplish one or more of the desired effects, plant extracts can be preserved in their health-promoting characteristics, as they are encapsulated in a matrix or membrane, the particulate state. Encapsulation is used to improve the stability of extracted chemicals during processing, storage, or transportation. Furthermore, the primary goal is to convert liquid chemical compounds into solid forms in order to improve active component management. The use of pharmaceutical formulations with encapsulated compounds as delivery systems can be particularly beneficial in situations where the direct consumption of an active ingredient directly disrupts the human skin. Encapsulation can also be used to improve the final product’s quality, separate incompatible chemicals, and administer bioactives in a regulated manner [10].

Enclosed vesicles known as liposomes are created when lipid components, including phospholipids, are distributed across an aqueous media. The inner water phase is divided from the outer one by one or more produced bilayers with a structure similar to the cell membrane [11]. Due to their unique structure, liposomes provide a number of significant advantages when used in drug delivery systems. Initially, the capacity of the contained vesicles to differentiate between the inner and exterior phases improves the stability of the encapsulated medicine. Second, when poorly hydrosoluble medicines are integrated into liposomes, their bioavailability improves [12,13]. Furthermore, following encapsulation, a regulated or sustained drug release profile may be obtained. Moreover, liposomes have outstanding biodegradability and a strong affinity for cells. Additionally, the target effect of liposomal preparations has the ability to alter the loaded drug’s in vivo distribution, hence increasing the medicinal index of some medications [14,15]. Furthermore, the surface of the liposomes may be altered in a number of ways, affecting the ultimate product’s characteristics and biological activity. Liposomes are small, spherical particles composed mainly of lipid mixtures, organized into one or more lipid bilayers. This type of organization allows for their use as a simple approximation to living cells [16]. Liposomes can be a highly valuable tool for identifying the fundamental interactions of bioactive substances with lipid bilayers since they are fairly simple to make, somewhat stable, and considerably less delicate to handle than human cell lines. To be precise, the encapsulation of bioactive compounds into nanoparticles or liposomes could decrease their toxicity, while increasing bioavailability, and also improve pharmacokinetics, thus leading to a better controlled release profile, and enhanced stability and solubility of the compounds in the organism [17]. This has led to a large number of areas for their possible medicinal applications, such as a source of treatment for cancer [18,19,20], skin disorders [21,22], post ischemia [23], pulmonary hypertension [24], etc. The development of liposomes intended for clinical use relied on the development of methods that would allow for the rapid formation of homogeneous small liposomes and efficient loading of drugs or bioactive compounds into them [25]. This was achieved by employing the extrusion technique and/or changes in the pH value of the mixture. 

Biopolymer films and coatings are among the active technologies used to create patches for transdermal administration, providing extra protection of biologically active components in order to retain or increase the overall quality of bioactives from plants and extend their shelf life [26,27]. Plant extracts high in polyphenols have been demonstrated to improve the antioxidant activity, UV light barrier capacity, and oxygen barrier ability of biopolymer films, hence enhancing the preservation of biologically active components [28,29]. Polyphenol interactions with the biopolymeric matrices may alter the structural and functional properties of the matrix [29,30], as well as a decrease the bioactivity of polyphenols; thus, potential interactions and their influence on the biological activities of polyphenols should be thoroughly investigated. 

Biologically active compound from plants have been subjected to various encapsulation methods in the past, which could be divided into three larger groups: physical (extrusion, freeze drying, supercritical fluid, pan-coating, electrospinning, etc.), chemical (inclusion complexation, emulsion polymerization, interfacial polymerization), and physicochemical methods (including coacervation, the sol-gel method, solvent evaporation, and similar methods) [31]. A less explored alternative method for the antioxidant polyphenol encapsulation is the combination of biopolymer films and liposomes. The active ingredients in this combination may be shielded from processing, storage, and environmental factors [32], preserving their bioactive qualities and allowing for controlled release and prolonged shelf life.

Proliposome technology offers a higher energy input of agitation, resulting in smaller and more homogeneous liposomes [33,34]. Using this method might be the easiest way to obtain liposomes [16]. The main drawback is that although it produces a substantially better encapsulation efficacy, it is not as reproducible when producing smaller amounts of liposomes. During the process of the making of liposomes, the compounds can be combined with ethanol (in the case of lipophilic substances) or in an aqueous solution (in the case of hydrophilic substances). The use of the proliposome method presents a chance for large-scale liposome production [35].

Wet processing, also known as solvent casting, is based on the drying process of the film-forming solution, which involves the phases of solubilization, casting, and drying [36]. Firstly, a biopolymer is dissolved in a suitable solvent, organic or inorganic, in order to create a film-forming solution. However, water, ethanol, or their combinations are the only medical-grade solvent systems available for biopolymer films and coatings. The dissolved film-forming solution may be heated or its pH adjusted on occasion to improve film formation or its properties. After that, the solution is dried by being cast onto a flat surface to form a film matrix [37]. The development of a continuous three-dimensional network among biopolymers is critical for the construction of a cohesive film [38]. The nature, kind, and amount of the interaction are determined by the polymers involved and film-forming factors such as drying temperature and pace, moisture content, solvent type, plasticizer concentration, and pH.

The rheological, physical, and chemical characteristics of topical drug delivery systems impact drug bioavailability and the production of pharmaceutical and cosmetic formulations. Therefore, it is imperative to investigate the aforementioned characteristics of liposomes and films. The study of the interactions between bioactive substances, medications, vitamins, or hormones with phospholipid liposomal bilayers is frequently carried out using Fourier transform infrared (FT-IR) spectroscopy [39,40].

The encapsulation of the petal extracts of Serbian *P. tenuifolia* into liposomal particles and biopolymer films has not been the focus of any previous studies. As a result, the goal of this study was to create and characterize extract-loaded liposomes, as well as extract- and liposome-loaded films in order to potentially protect the sensitive biologically active components, increase their bioavailability, and enable controlled release, making them suitable for use in a variety of pharmaceutical and cosmetic formulations.

## 2. Materials and Methods

### 2.1. Origin of Plant Material

**Figure 1:**
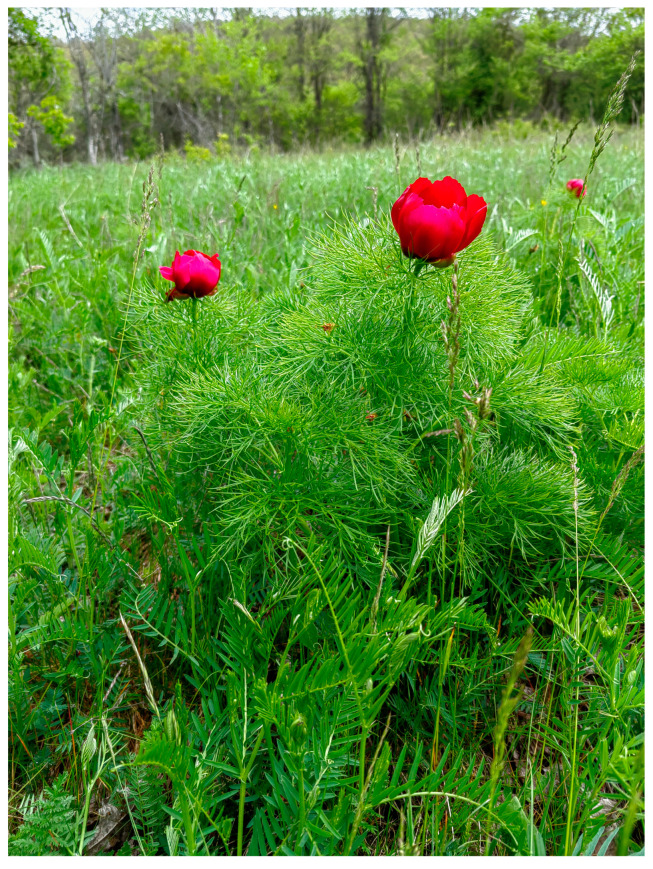
Full-blooming *Paeonia tenuifolia* L. at the locality Gulenovci, Serbia (May 2023).

*P. tenuifolia* L. fresh petals were gathered in May 2023 from plants growing spontaneously in their native habitat in Gulenovci (840 m a.s.l.), Serbia (Figure 1). The Ministry of Environmental Protection of the Republic of Serbia granted the license for wild-collecting (no. 353-01-121/2023-04, issued on 3 March 2022). The petals were collected by hand from randomly selected full-blooming flowers. A third of the petals per flower were taken from less than one-tenth of the flowering plants found at the locality. Before undergoing the extraction processes, the gathered petals were shade-dried at room temperature.

### 2.2. Extraction of Plant Material

The biologically active compounds of the petals were extracted using the maceration process, by employing a linear mechanical homogenizer (Roller mixer SRT6, Potsdam, Germany) at room temperature (25 ± 5 °C) for 24 h using methyl alcohol as an extraction medium, with a solid-to-solvent ratio of 1:20. The extracts from the petals were filtered using laboratory filter paper. Before further analysis, the collected extract was evaporated at 30 °C in a drying oven (Sanyo drying oven MOV-212, Eschborn, Germany) to a dry mass, and kept in the dark at 4 °C.

### 2.3. Preparation of Liposomal Particles 

The liposomes containing *P. tenuifolia* L. petal extract were prepared utilizing the proliposome technique [34]. The liposomes were prepared using three mixtures of phospholipids: Phosal SA 75 (containing phosphatidylcholine in ethanol and safflower oil, content ≥ 72.0%), Phosal MCT 53 (phosphatidylcholine in medium-chain triglyceride, content ≥ 53.0%) (from Lipoid, Skopje, Macedonia), and Phospholipon (a commercial lipid mixture, sunflower phosphatidylcholine from non-genetically modified plants, ≥90%; from Lipoid GmbH, Ludwigschafen, Germany). At 50 °C, the phospholipids (4 g), ethanol (15 mL), deionized water (3 mL), and dried *P. tenuifolia* L. petal extract (0.4 g) were mixed. After the emulsion had cooled to room temperature, the aqueous phase (20 mL) was added in tiny amounts and the emulsion was agitated at 800 rpm for 1 h. As a control, plain liposomes were also made.

### 2.4. Preparation of Liposome-Loaded Films

Carboxymethyl cellulose (CMC) (0.48 g) (medium viscosity, 400–800 cP, a molar mass of 250 kDa, approximately, degree of substitution between 0.65–0.9; from Sigma Aldrich, Hamburg, Germany), deionized water (12 mL), and propylene glycol (0.36 mL) were mixed with a magnetic stirrer at 500 rpm for 24 h at room temperature. The resultant mixture was put into Petri plates and dried for 24 h in a laboratory oven (Emmeret UN 160, Emmeret GmbH + Co. KG, Schwalmstadt, Germany). Finally, the biopolymer films were conditioned at room temperature for three days in a desiccator containing magnesium sulfate.

### 2.5. Preparation of Extract-Loaded Films

CMC (0.96 g), deionized water (24 mL), and propylene glycol (0.36 mL) were added to the extract solution (0.4 g of dried petal extract in 12 mL of deionized water) and mixed for a whole day at room temperature with a magnetic stirrer at 500 rpm. The resulting mixture was put onto Petri plates and dried for twenty-four hours in a laboratory oven (Emmeret UN 160, Emmeret GmbH + Co. KG, Schwalmstadt, Germany). The biopolymers were kept the same way as the liposomes. The film type, CMC-0.2 ex, was created using the same technique, but with the addition of 0.2 g of the dried petal extract.

### 2.6. Encapsulation Efficiency

The liposome–extract particles were separated from the non-encapsulated fraction via centrifugation at 17,500 rpm and 4 °C, for 45 min (Thermo Scientific Sorval WX Ultra series ultracentrifuge, ThermoScientific, Waltham, MA, USA). The encapsulation efficiency (EE) was determined by measuring the total polyphenol content (TPC) in the supernatant using UV-Vis spectrometry (Shimadzu 1800 UV/Vis spectrophotometer, Kyoto, Japan), and the Folin–Ciocalteu method previously described by Čutović et al. [3]. EE was calculated according to the amount of polyphenols present in the supernatant, obtained after centrifugation as shown in Equation (1):

(1) $$EE\left[\%\right]=\frac{{TPC}_{i}-{TPC}_{sup}}{{TPC}_{i}}\times 100\%$$

TPC_i_ denotes the initial total polyphenol content of the *P. tenuifolia* L. petal extract utilized for liposome synthesis, whereas TPC_sup_ denotes the total polyphenol content found in the supernatant after centrifugation.

### 2.7. Fourier Transform Infrared Spectroscopy (FT-IR)

The chemical interactions between the film components, extract compounds, as well as the liposome ones for all prepared liposomes and films were characterized via FTIR spectroscopic analysis. The FTIR spectra were collected using a Nicolet iS10 ATR-IR spectrometer (Thermo Scientific, Stockholm, Sweden) with a scanning resolution of 4000 cm^−1^. For the FTIR measurements, polymer biofilms were sliced into tiny plates (1 × 1 cm) and mounted on the Diamond chassis. The liposomes were lyophilized (empty and extract-loaded), and the process consisted of centrifugation, after which the liposomes were frozen in the freezer, LAB11/EL19LT (Elcold, Hobro, Denmark), at −80 °C for 1 h and lyophilized in Beta 2–8 LD plus lyophilizator (Christ, Memmingen, Germany) at −75 °C and 0.011 mbar for 24 h.

### 2.8. Moisture Content

The moisture content was calculated by weighing each film sample after oven drying to a constant weight at 30 ± 0.2 °C. The moisture content was calculated according to Equation (2):

(2) $${MC}_{wb}=\frac{m_{1}-m_{2}}{m_{1}}\times 100\%$$

where m_1_ stands for the initial weight (g) of the samples, and m_2_ and MC_wb_ are the dry weight (g) and wet basis moisture content of the samples, respectively.

### 2.9. Stability Study

The size of the particles, PDI, and ζ potential of the prepared liposomes were measured every 7th day for the first 28 days, and the on the 60th day, following preparation using the Malvern Zetasizer Nano ZS (Malvern Instruments, Worcestershire, UK). During the 60-day stability evaluation, the liposomal system was stored in the refrigerator at 4 °C.

### 2.10. Rheological Characteristics

The density and surface tension of petal extract-loaded liposomes were measured in a Force Tensiometer K20 (Kruss, Hamburg, Germany) using a silicon crystal as the immersion body and a Wilhelmy plate, respectively. At 25 °C, each sample (20 mL) was analyzed three times.

Using a Rotavisc lo-vi device equipped with a VOL-C-RTD chamber, VOLS-1 adapter, and spindle (IKA, Staufen, Germany), the viscosity of petal extract-loaded liposomes was measured. At 25 °C, each sample (6.7 mL) was analyzed three times.

The density, surface tension, and viscosity measurements were taken on the first and sixtieth day.

### 2.11. Determination of the Film Mechanical Properties

**Figure 2:**
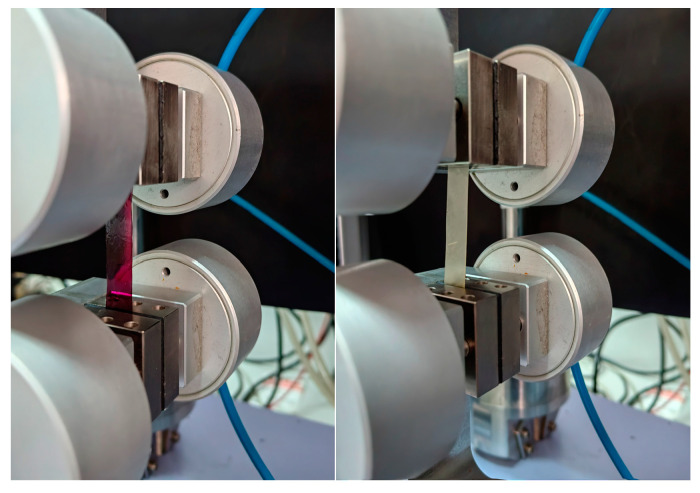
Tensile testing of the carboxymethyl cellulose-based films using a Universal Testing Machine; sample between the grips containing the petal extract (**left**) and extract-loaded liposomes (**right**).

The tensile strength (TS, MPa), break force (BF, N), and elongation at break (EB,%) of the films were measured with a Universal Testing Machine (Shimadzu Corporation, Kyoto, Japan) outfitted with a 100 N load cell (Figure 2). The rectangular film strips (25 × 80 mm) were stretched at a crosshead speed of 10 mm/min using stainless steel grips. The gage length was measured as 50 mm. The measurements were obtained three times. The mechanical properties of the films were determined using engineering stress–strain and force–displacement curves at the breaking point [26]. The film thickness was measured using a digital nonius depth caliper (0-Industrial&Scientific, Pittsburgh, PA, USA, 0–150 mm), and the film weight was determined using an analytical balance (Mettler, Columbus, OH, USA, Type AE 200; 0.0001 g). 

### 2.12. Biological Activities

The assessed biological activities of the encapsulated extracts (in liposomal, film, and liposome–film systems) included their antibacterial and anticandidal, as well as antibiofilm activities, although the antibiofilm activity was performed only for liposomes. 

#### 2.12.1. Antibacterial Activity of Liposomal Particles

Gram-positive bacteria (*Staphylococcus aureus* ATCC 11632 and *Staphylococcus lugdunensis* Ibis 2996) and Gram-negative bacteria (*Proteus vulgaris* IBR P004) were examined for antibacterial activity with liposomes. The microdilution technique (96-well microtiter plates) was used to determine the minimum inhibitory concentration (MIC) and minimum bactericidal concentration (MBC), as reported before [41]. The samples were placed in a tryptic soy broth (TSB) medium and infected with bacteria at a final concentration of 1 × 10^6^ colony-forming units (CFU) per well. Gentamicin (Panfarma, Belgrade, Serbia) was used as the positive control. As the negative control, blank liposomes were utilized. The MIC and MBC values were provided in milligrams per milliliter.

#### 2.12.2. Antibacterial Activity of Liposome- and Extract-Loaded Films

The antibacterial activity of the liposome- and extract-loaded films was determined using the disc diffusion assay [42]. Inoculums of the test bacteria were produced in the same way that overnight bacterial cultures were. On a Mueller-Hinton agar plate, 300 μL inoculums were used to create uniform bacterial lawns. In the center of the Petri dish, thin film squares (10.0 × 10.0 mm) were inserted. The plates were incubated for 24 h at 37 °C. The zone of inhibition (mm) was used to quantify activity. The net zone of inhibition was calculated by subtracting the square side (i.e., 10.0 mm) from the overall zone of inhibition demonstrated by the test disc in terms of the clear zone surrounding the disc. As the positive control, streptomycin was utilized. As the negative control, blank films were utilized.

#### 2.12.3. Anticandidal Activity of Liposomal Particles

*Candida albicans* (Y177), *Candida kefyr* (Y289), and *Candida krusei* (Y454) were used in order to test the extract-loaded liposome antifungal activity. The modified EUCAST protocol (EUCAST, 2002) was used to carry out the anticandidal assay, as previously explained [43]. The positive control was ketoconazole, while the negative control was blank liposomes. The MIC and MFC (minimum fungicidal concentration) values are presented as mg/mL.

#### 2.12.4. Anticandidal Activity of the Liposome- and Extract-Loaded Films

Anticandidal activity of the liposome- and extract-loaded films was determined using the disc diffusion assay [42]. Inoculums for testing *Candida* strains were produced in the same way that overnight fungal cultures were. On a TSB plate, uniform *Candidal* lawns were created using 300 μL inoculums. In the center of the Petri dish, thin film squares measuring 10.0 × 10.0 mm were inserted. The plates were incubated for 24 h at 37 °C. The zone of inhibition (mm) was used to quantify activity. The net zone of inhibition was calculated by subtracting 10.0 mm from the overall zone of inhibition revealed by the test disc in terms of the clear zone around the disc. As the positive control, ketoconazole was utilized. As the negative control, blank films were utilized.

#### 2.12.5. Antibiofilm Activity of Liposomal Particles

The petal extract-loaded liposome effect on the *S. lugdunensis* biofilm was assessed with minor alterations, as previously described in Smiljkovic et al. [44]. *S. lugdunensis* was grown in TSB with 2% glucose on 96-well microtiter plates with adhesive bottoms (Sigma Aldrich, Taufkirchen, Germany), with MIC, MIC/2, and MIC/4 of the extract-loaded liposomes for 24 h to form a biofilm. After incubation, the wells were washed twice with sterile PBS (phosphate buffer solution). The biofilm was then treated with methyl alcohol and air-dried. For 30 min, the biofilm was stained with crystal violet (Bio-Merieux, Crappone, France). After incubation, the crystal violet was removed, the wells were cleansed with water, air-dried, and then 96% ethanol (Zorka, Šabac, Serbia) was used. Thermo Fisher Scientific’s Multiskan FC Microplate Photometer (Waltham, MA, USA) was used to detect absorbance at 620 nm, and Equation (3) was used to calculate the percentage of biofilm suppression. Gentamicin (Panfarma, Belgrade, Serbia) was employed as the positive control, whereas blank liposomes were used as the negative control.

(3) $$\%\;of\;biofilm\;inhibition=\frac{A_{620,\;control}-A_{620,\;sample}}{A_{620,\;control}}\times 100\%$$

### 2.13. Release Study

Using the Franz diffusion cell (PermGear, Inc., Hellertown, PA, USA), studies of the controlled release of polyphenols from the extract, liposomes, liposome-loaded films, and pure extract-loaded films were carried out. The donor and acceptor compartments of a Franz cell are divided by a cellulose acetate membrane (Permgear, Hellertown, PA, USA) with a diffusion area of 4.91 cm^2^ and a pore size of 0.2 µm. The donor compartment (0.05 g, d = 2.5 cm) received the samples. A magnetic stirrer was used to continuously mix the release media (phosphate buffer, pH = 5.5, c = 0.1 mol/L) at 37 °C and 400 rpm in the receptor compartment [39]. For 24 h, samples were collected at set times. The Folin–Ciocalteu method, as previously indicated by Čutović et al. [3], was adjusted for the quantification of the polyphenols in these samples. Briefly, 20 μL of the controlled release sample, 100 μL of the Folin–Ciocalteu reagent, and 1500 μL of deionized water were placed in a 2000 μL flask. Subsequently, 300 μL of sodium carbonate (20% *w*/*v*) was added, and the mixture was then topped off to a volume of 2000 μL with deionized water. After 120 min of incubation at room temperature and in the dark, the absorbance at 765 nm was measured. The results of each analysis were statistically processed after being run three times.

### 2.14. Scanning Electron Microscopy (SEM) 

The morphology of the biopolymer films containing the *P. tenuifolia* petal extract or liposomes was evaluated using scanning electron microscopy (Tescan Mira3 XMU, Cranberry Township, PA, USA), operated at 10 keV. The samples were subjected to analysis in their dry forms. Prior to SEM analysis, all three samples of the films were cut on a tile (5 × 5 mm), fixed on a sample holder, and then vacuum-coated with a gold/platinum alloy (15/85) using a Polaron SC502 vacuum sputter coater. 

### 2.15. Statistical Analysis

The statistical analysis in this study was carried out using an analysis of variance (one-way ANOVA) followed by Duncan’s *post hoc* test inside the statistical program STATISTICA 7.0 (TIBCO Software Inc., Palo Alto, CA, USA) [45]. Duncan’s multiple range test is a statistical test known for its use in order to compare the means of multiple groups of results. It is a post hoc test that is performed after the analysis of variance (ANOVA) test reveals a significant difference. The test detects whether group means differ significantly from one another, and divides them into subgroups depending on their similarities. The differences were considered statistically significant at *p* < 0.05, n = 3.

## 3. Results

This study looks at the liposomes’ encapsulation effectiveness, particle size, index of polydispersity (PDI), ζ potential, density, surface tension, viscosity, antibiofilm activity, and 60-day storage stability. The mechanical characteristics and moisture content of the films were determined. Furthermore, all the liposomes and films were examined for antibacterial and antifungal activities, as well as controlled release studies and FT-IR chemical composition analysis.

### 3.1. Encapsulation Efficiency

**Figure 3:**
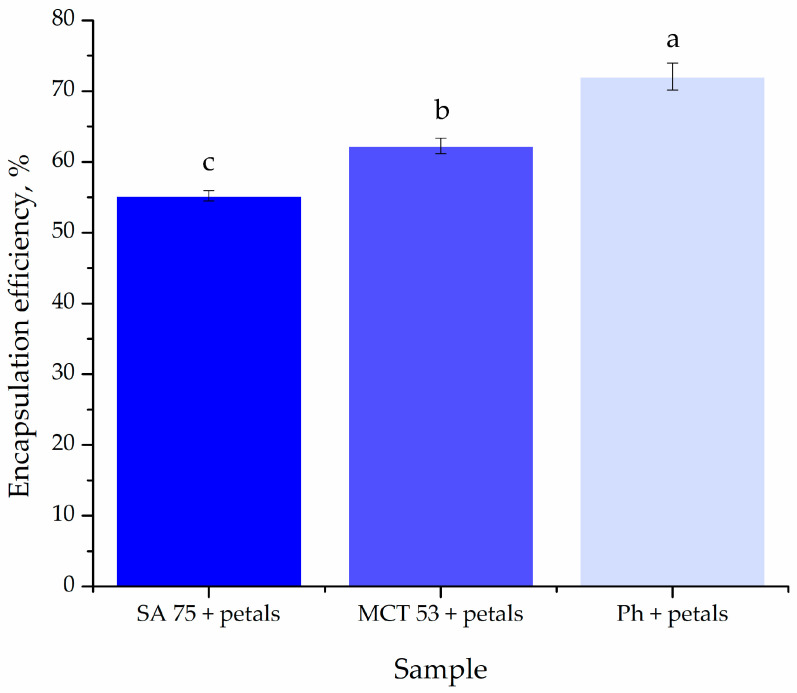
Encapsulation efficiency of *Paeonia tenuifolia* L. petal extract-loaded liposomes; different letters show a statistically significant difference (*p* < 0.05; n = 3, one-way ANOVA, analysis of variance, Duncan’s *post hoc* test).

The encapsulation efficiency of *P. tenuifolia* L. petal polyphenols inside liposomes was investigated and is shown in Figure 3 since it is one of the most significant parameters specified by the encapsulation process [46]. The effectiveness of encapsulation was assessed right after the preparation of the liposomes.

The encapsulation efficiency of *P. tenuifolia* L. extracts into three different types of liposomes was, respectively, 55.20 ± 0.70 for SA 75, followed by MCT 53 at 62.26 ± 1.1%, making Ph liposomes the most efficient for encapsulating *P. tenuifolia* L. petal bioactive compounds, with an efficiency level of 72.04 ± 1.9% (Figure 3). 

### 3.2. FT-IR Spectra

**Figure 4:**
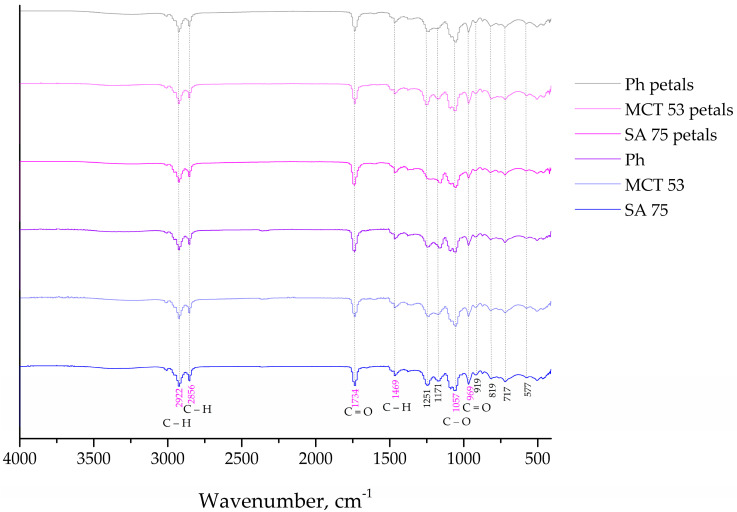
FT−IR spectra of the plain and *Paeonia tenuifolia* L. petal extract-loaded liposomes.

**Figure 5:**
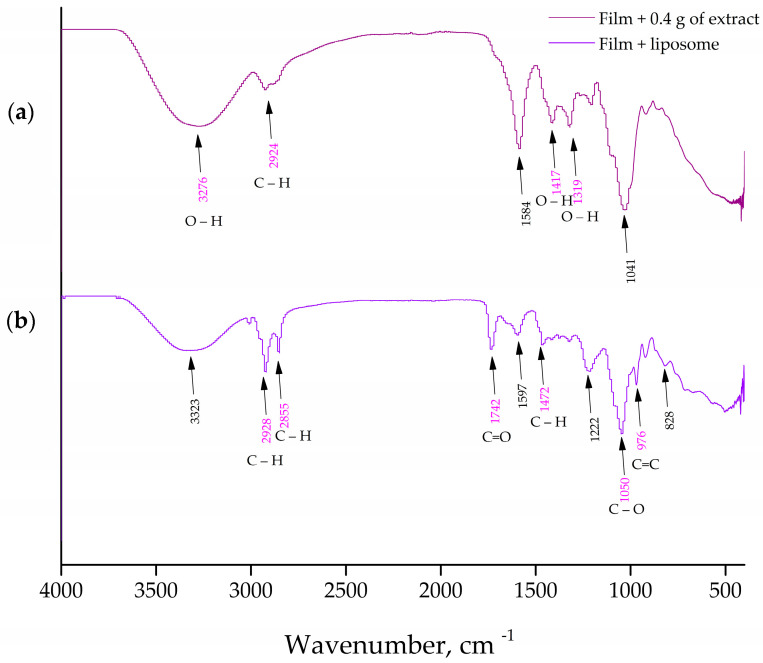
(**a**) FT-IR spectra of a *Paeonia tenuifolia* L. petal extract-loaded carboxymethyl cellulose film; (**b**) FT-IR spectra of a liposome-loaded carboxymethyl cellulose film.

The FT-IR spectroscopy was used to analyze the presence of distinct interactions between the phospholipids from the liposomes and the carboxymethyl cellulose from the films containing the *P. tenuifolia* L. flower extract. Figure 4 and Figure 5 show the FT-IR spectra of blank liposomes, extract-loaded liposomes, and extract- and liposome-loaded films.

### 3.3. Moisture Content

Given that it influences the film’s mechanical strength, adhesive qualities, and friability, the moisture content of the material may be of great importance. The hygroscopic qualities of active medicinal compounds, polymers, the solvent system used to dissolve the polymeric mixture [47], and manufacturing processes are some of the reasons that cause the water level to rise, which is why the moisture content of the films containing only extract and extract-loaded Ph liposomes was subjected to the assessment of it. The moisture content of the films ranged from 92.38 to 94.73% (Figure S1). The control films, containing only pure extracts, had a slightly higher moisture content than the one containing the extract-loaded liposomes. 

### 3.4. Stability Study

**Figure 6:**
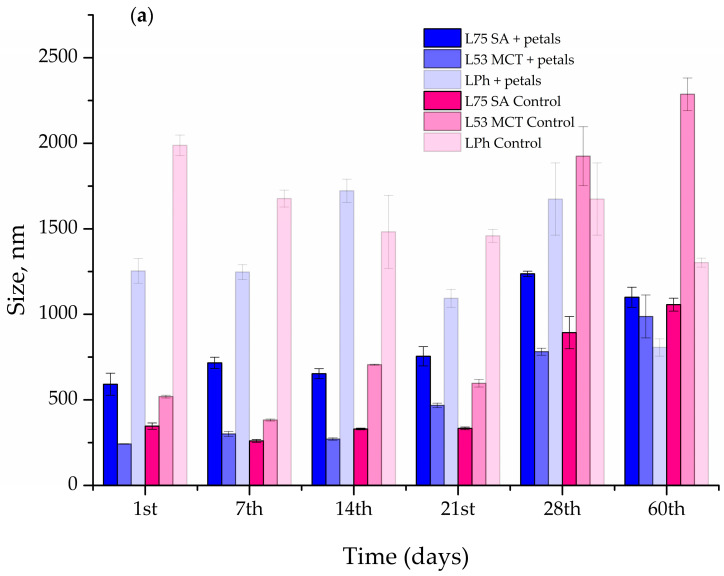

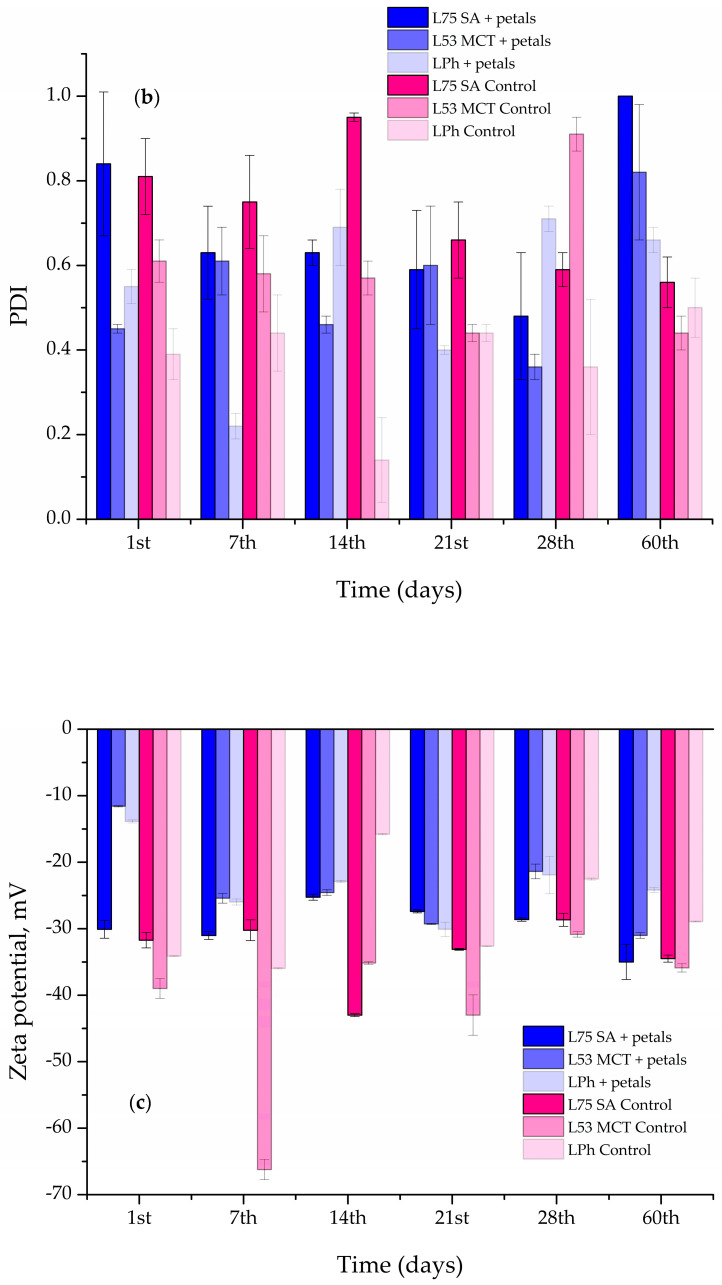
Liposome size (**a**), polydispersity index (PDI) (**b**), and zeta-potential (**c**) of the blank and *Paeonia tenuifolia* L. petal extract-loaded liposomes during 60 days of storage at 4 °C.

Particle size, PDI, and ζ potential were evaluated for 60 days to test the storage stability of the blank and *P. tenuifolia* L. petal extract-loaded liposomes, and the findings are shown in Figure 6a–c.

In the case of liposomes L75 SA and L53 MCT, the size of the liposomes containing the *P. tenuifolia* L. petal extract did not change appreciably after 14 days of storage at 4 °C (Figure 6a). After the 21st day, the size of the vesicles increased from 653.13 ± 29.25 nm to 754.7 ± 56.38 nm for the L75 SA liposomes as the lipid component, and amounted to 1100 ± 58.53 nm on the 60th day. The PDI continuously decreased until the 28th day (Figure 6b), and then exponentially rose at the 60-day mark. In the case of the liposomes comprised of L53 MCT and the petal extract, the size of the particles changed significantly after 21 days of storage and increased from 242.53 ± 0.61 to 467.70 ± 12.87 nm, reaching a size of 987.50 ± 125.93 nm after 60 days. The PDI varied during the 60 days of storage, as it had decreases and increases in its value during this period. The liposomes that had the biggest vesicles were the ones prepared using Phospholipon and *P. tenuifolia* L. petal extract, as the starting size of the vesicles was 1555.33 ± 24.28 nm. It increased up to the 14-day mark, reaching 1722.00 ± 68.19 nm, and then slowly decreased in size, coming down to 806.23 ± 50.50 nm after 60 days. In the case of the PDI, it had an unsteady trend, similar to the L53 MCT liposomes, as it had increases and decreases in value throughout the period of storage. 

As can be seen in Figure 6a, on the 1st day of the storage stability study, the blank L75 SA liposomes were noticeably smaller than the extract-loaded parallel, whereas the L53 MCT and LPh liposomes followed a similar trend as their extract-loaded analogs. The vesicle diameters of all blank liposomes altered after 60 days of storage at 4 °C, but the L75 SA and L53 MCT ones increased in size, whereas the LPh liposomes became smaller. The most significant change in the size after the 60-day period at 4 °C can be seen in the L53 MCT control liposome, as it increased by 77.29%. 

A negative zeta-potential implies that the phospholipid molecules are organized, whereas high values suggest that the liposomal suspension is electrostatically stabilized, making particle aggregation slower and longer, and therefore the liposomes persist longer [34].

Moreover, the zeta-potential varied in both the blank and petal extract-loaded liposomes, as can be seen in Figure 6c. The zeta-potential of L75 SA liposomes with petal extract did not significantly change up to the 28th day, changing from −30.10 ± 1.34 mV and exponentially increasing to −35.00 ± 2.65 mV (increase in the absolute value). The L53 MCT and LPh liposomes containing the petal extract showed an unsteady trend in their stability, as their zeta-potential had both increases and decreases in its value during the storage period of 60 days. In the case of the blank liposomes, the one that stood out the most is L53 MCT, which had a huge spike in the decrease in the zeta-potential at the 7th day mark, with a value decrease of 50.37%, after which it increased up to the 60-day mark, reaching −35.90 ± 0.62 mV.

Since the encapsulation of extract compounds can significantly affect the zeta-potential of the liposomal suspension, the mentioned parameter was measured for pure extract as well. The zeta-potential of the petal extract amounted to −15.9 ± 0.9 mV.

### 3.5. Rheological Characteristics and Physical Properties of Extrac-Loaded Liposomes

Table 1 shows the rheological parameters of *P. tenuifolia* L. petal extract-loaded liposomes, which include density, surface tension, and viscosity. Because the aforementioned characteristics play an important role in the production of pharmaceutical and cosmetic goods, they should be examined in order to improve product quality.

As seen in Table 1, the density of the extract-loaded liposomes ranged from 1.031 to 1.066 g/cm^3^ on the 1st day, whereas the mentioned parameter increased after 60 days, ranging from 1.098 to 1.151 g/cm^3^. The surface tension of the *P. tenuifolia* L. petal extract-loaded liposomes decreased from ~22.5 mN/m, to a range from 9.23 to 19.57 mN/m after a 60-day storage period at 4 °C. The viscosity of the petal extract-loaded liposomes right after they were prepared was in the range of 96.97 to 268.40 mPa∙s depending on the lipid used as the base for the liposome preparation, whereas after the 60-day storage of the liposomes, their viscosity changed, spanning 101.57 to 329.97 mPa∙s.

### 3.6. Mechanical Properties of the Films 

The break force, elongation, and tensile strength of the films with extract and extract-loaded liposomes were determined with the aim of defining the mechanical stability of the coatings that could be of significance for their further application.

Table 2 shows the mechanical characteristics, tensile strength (TS), break force (BF), and elongation at break (EB) of the CMC films. It can be seen that the film containing 0.4 g of *P. tenuifolia* L. petal extract can withstand the highest force, before reaching its breaking point, being 12 times tougher than the film containing LPh liposomes. 

### 3.7. Biological Activities

#### 3.7.1. Antibacterial Activity of Liposomes

Table 3 displays the results of the antibacterial efficacy of plain and *P. tenuifolia* L. petal extract-loaded liposomes against *S. aureus*, *P. vulgaris*, and *S. lugdunensis* naturally present on human skin.

The antibacterial activity of the plain liposomes and liposomes loaded with the extract of the *P. tenuifolia* L. petals was tested against three bacterial strains: *S. lugdunensis*, *S. aureus*, and *P. vulgaris* (Table 3). The empty liposomes were used as a control for the influence of lipids on the antibacterial activity, and they showed that the most promising one is the L53 MCT, followed by a slightly less effective LPh. On the other hand, the liposomes made with Phospholipon as the substrate demonstrated the best antibacterial action, being the most effective against *S. aureus* and *P. vulgaris* (MIC 0.5 mg/mL). *S. lugdunensis* had the greatest resilience to the impact of the petal extract-loaded liposomes. (MIC 0.5–4 mg/mL). The most efficient liposomes against *S. aureus* and *P. vulgaris* (MIC 0.5 mg/mL) were those loaded with the extract. 

#### 3.7.2. Antifungal Activity of Liposomes

The obtained liposomes with the *P. tenuifolia* L. petal extract were initially tested against fungi found on human skin, *C. albicans*, *C. kefyr*, and *C. krusei*. The MIC value is defined as the lowest concentration of antifungal agent required to inhibit fungus growth by serial dilution, whereas the MFC value represents the least concentration of biologically active chemicals required to prevent pathogen development.

The antifungal efficacy of liposomes was evaluated against three therapeutically significant *Candida* species that are commonly detected on human skin (Table 4). In parallel trials, ketoconazole was utilized as a typical antifungal treatment to control the susceptibility of the investigated fungus strains. The antifungal activity of all *P. tenuifolia* L. petal extract-loaded liposomes against the tested *Candida* strains was moderate. However, the most promising antifungal effect was achieved against *C. krusei*, when the Phospholipon was used as the lipid for the preparation of the petal extract-loaded liposomes. The liposome MIC values against *C. krusei* ranged from 0.25 to 4 mg/mL, whereas the MFC values ranged from 0.5 to 8 mg/mL. Liposomes were the least effective in slowing the growth of *C. kefyr*, as the MIC values had gone up to 8 mg/mL. The antifungal activity of the tested liposomes decreased in the following order: *C. krusei* > *C. albicans* > *C. kefyr*. 

#### 3.7.3. Antibacterial Activity of Liposome- and Extract-Loaded Films

The antibacterial activity of extract-loaded and liposome-loaded films was studied against both Gram-positive (*S. aureus*, and *S. lugdunensis*) and Gram-negative bacteria (*P. vulgaris*). Table 5 displays the results of the antibacterial screening of the films. 

The zones of inhibition were detected in the disk diffusion test and were in the range of 7.5 mm–30.165 mm (Table 5). The most sensitive bacteria were *P. vulgaris* with an inhibition zone of 30.165 mm, followed by *S. aureus* with an inhibition zone of 27.833 mm, when tested for the influence of the extract-loaded film containing 0.4 g of the *P. tenuifolia* L. petal extract. The thin film with the smallest diameter of the inhibition zone, was the one containing liposomes, as it had no activity against *S. aureus* and *S. lugdunensis*, and very little activity against *P. vulgaris*. 

#### 3.7.4. Antifungal Activity of Liposome- and Extract-Loaded Films

The obtained thin films were subjected to the testing of their antifungal activity against two *Candida* strains (*C. kefyr* and *C. krusei*), and the diameters of the zones of the growth inhibition are presented in Table 6. *C. albicans* was also tested for the effect of films on the formation of uniform lawn, but this *Candida* species did not grow on the used plates, so the results for this strain were not obtained.

When the disc diffusion assay was conducted on the *Candida* strains of interest, the zones of inhibition varied from 10.33 mm to 35.16 mm (Table 6). The extract-loaded film showed an increase in their ability to inhibit the growth of the fungi as the amount of extract encapsulated in the films got higher. The zone of inhibition was the widest when the film containing 0.4 g of *P. tenuifolia* L. petal extract was used on the *C. kefyr* strain. 

#### 3.7.5. Antibiofilm Activity of Extract-Loaded and Empty Liposomes

**Figure 7:**
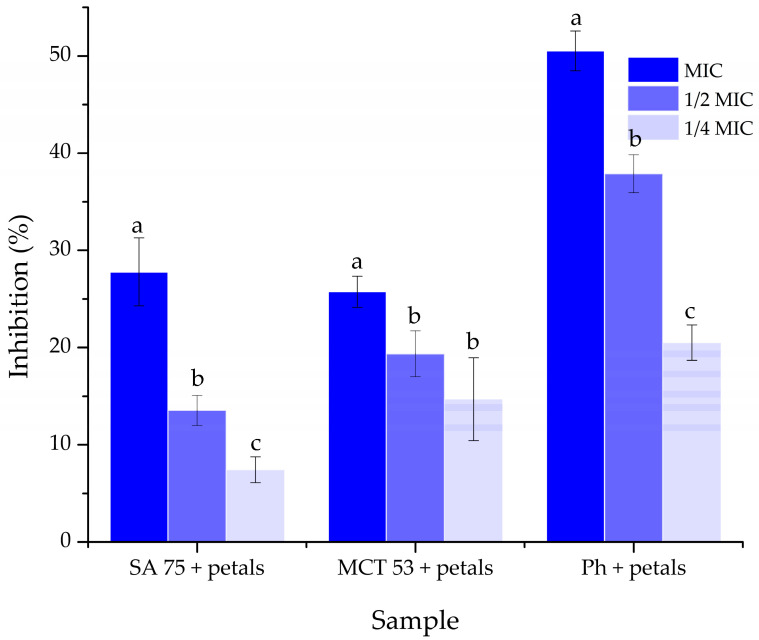
Biofilm formation of Staphylococcus lugdunensis (after treatment with *Paeonia tenuifolia* L. petal extract-loaded liposomes). Different letters in each group of data (SA 75 + petals, MCT 53 + petals, and Ph + petals) show a statistically significant difference (*p* < 0.05; n = 3, one-way ANOVA, analysis of variance, Duncan’s post hoc test).

The petal extracts that were incorporated into liposomes in this study have previously been tested for their antibiofilm activity against *S. lugdunensis* by our research group, which is why the petal extract-loaded liposomes were tested for their antibiofilm activity against this pathogen. The results are presented in Figure 7.

Because the extracts of the petals collected in Gulenovci demonstrated the best inhibitory activity against the bacterial strains of *S. lugdunensis* in our previous work [3], this bacterium was chosen for the evaluation of the antibiofilm activity of the petal extracts when encapsulated in liposomes. Figure 7 depicts the findings of this assay. In particular, the liposomes prepared using Phospholipon showed the highest level of inhibition of biofilm formation, with 50.52% of inhibition at a concentration equal to the MIC, followed by the liposomes containing SA 75, which were better than the least efficient by only 2%. The level of inhibition had noticeably dropped with the use of lower concentrations. 

### 3.8. Release Study

**Figure 8:**
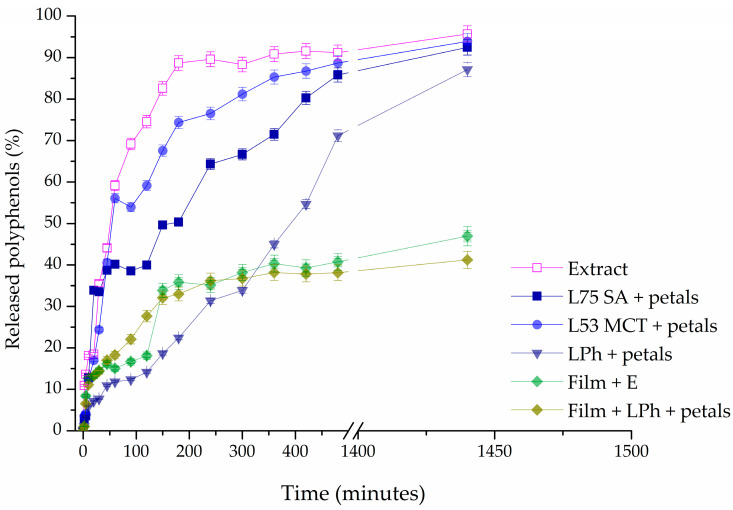
Release profiles of polyphenols from *Paeonia tenuifolia* L. extract, liposomes, and films, expressed as the percentage of released polyphenols.

A Franz diffusion cell was used to measure the mass transfer resistance of the liposomal and film membranes in the release studies of polyphenols derived from *P. tenuifolia* L. petals. In Figure 8, the values of the percentage of released polyphenols are displayed as a function of time during a 24 h period, illustrating the results. Using the same concentration of petal extract as that used to prepare the films and liposomes, the diffusion profile of the dry *P. tenuifolia* L. petal extract solution in ethanol served as a control for comparing the release of polyphenols from the films and liposomes. Liposomes containing SA 75, MCT 53, or Phospholipon as the lipid base loaded with *P. tenuifolia* L. petal extract, and CMC films containing 0.4 g of the petal extract, as well as the CMC film containing Phospolipon liposomes with the petal extract were selected to be examined.

The percentage of released polyphenols in the receptor counterpart presented in Figure 8 was calculated using the following equation:

(4) $$\%\;\mathrm{released}\;\mathrm{polyphenols}=100-\frac{m_{d}}{m_{d}^{0}}\times 100$$

where m_d_^0^ was the initial mass of total polyphenols in the samples (extract, liposomes, and films) in the donor counterpart and m_d_ was the mass of total polyphenols in the samples in the donor counterpart during time t.

The diffusion coefficients were estimated using the slope of the linear portion of the curve shown in Figure S3, whereas the diffusion resistance shows the ratio between the sample thickness and the diffusion coefficient. It can be seen in Table 7 that the LPh + petal liposome had the lowest diffusion coefficient among all of the tested liposomes, leading to the least mass of active components transferred from the donor to the acceptor part of the Franz diffusion cell. 

### 3.9. SEM Analysis of Biopolymer Films

**Figure 9:**
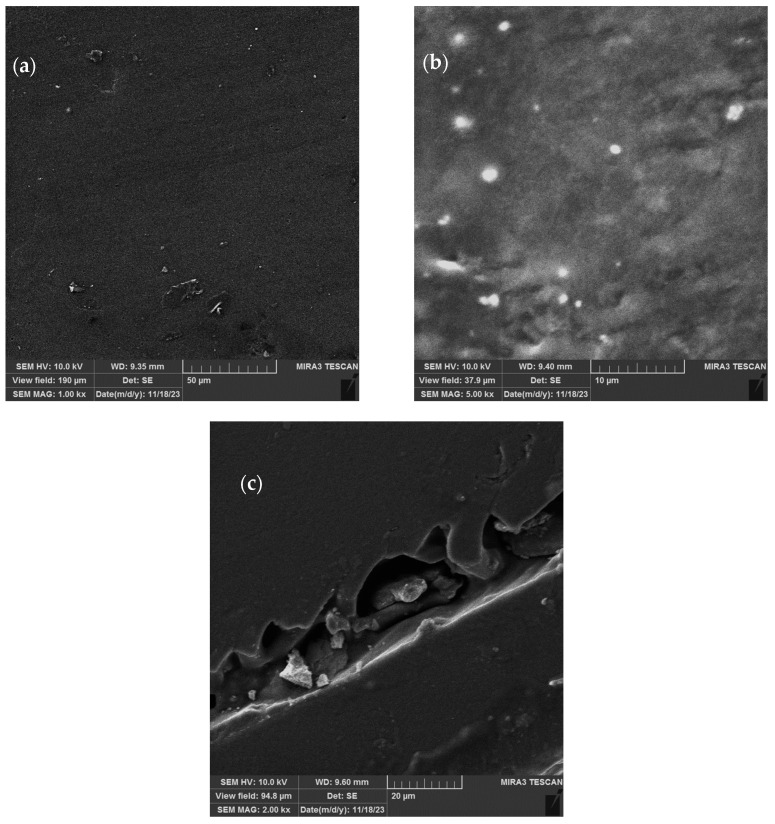
SEM micrographs of dried carboxymethyl cellulose films: (**a**) extract-loaded film (0.4 g) with low magnification (×1000); (**b**) liposome-loaded films with low magnification (×5000), and (**c**) extract-loaded film (0.2 g) with low magnification.

In order to provide more details about the material structure, the biopolymer films were subjected to SEM analysis. Figure 9a shows the surface of the extract-loaded films (0.4 g), where it can be seen that they are of uniform composition and compact; the shape and surface of the liposomes used for the encapsulation of the *P. tenuifolia* petal extract dispersed in the CMC films is presented in Figure 9b, while Figure 9c shows the cross-section of the films containing 0.2 g of the petal extract. Under low magnification (SEM MAG 1000–5000×), it can be seen that the extract-loaded polymer biofilms are of homogenous composition, while the liposomes that were embedded into carboxymethyl cellulose films (Figure 9b) have a globular and flattened structure.

## 4. Discussion

### 4.1. Encapsulation Efficiency

According to the literature, hydrophobic and hydrophilic bioactive substances may be encapsulated into lipid vesicles with an encapsulation effectiveness of 80 to 90%. However, the encapsulation efficiency of polyphenols from petal extracts in liposomes is often lower, at ~78% for butterfly pea (*Clitoria ternatea*) petal extract [48] and 64–87% for saffron (*Crocus sativus*) petal extract [49], depending on the used encapsulation method. In the present instance, a straightforward and affordable proliposome approach was used in order to successfully encapsulate *P. tenuifolia* L. polyphenols, which, according to the literature, include gallic acid, galloyl hexoside, ellagic acid, *p*-coumaric acid, and ferulic acid [3]. The proliposome method used in our study can be used to encapsulate both hydrophilic and hydrophobic bioactive compounds from various plant extracts due to the unique structure and makeup of the liposomal particles, which can entrap target compounds inside (in the surrounding water for hydrophilic compounds, or between phospholipid tails for lipophilic compounds) [50]. The difference in encapsulation efficiency between the liposomes prepared using Phospholipon (a solid phospholipid mixture) and those with Phosal phospholipids (liquid mixture) could possibly be due to the rigidity of the membrane. Phospholipon is a mixture of only lipids, which leads to a more rigid membrane; thus, the entrapment of the polyphenols in its structure is more stable [51], while the release of polyphenols from the liposomes containing the oil phase, besides the lipids, is easier.

### 4.2. FT-IR Spectra

Regarding the FT-IR spectra of the blank (control) liposomes (Figure 4), a group peak at 576 cm^−1^ assigned to O–CO–C of the present phospholipids, C–N symmetric stretching of choline at ~721 cm^−1^ is observed, and at 966 cm^−1^, there is a peak characteristic for the alkene stretching which is related to the trans disubstituted of the phenyl group, respectively [39,40]. There is a band at 1057 cm^−1^ that represents the C–O–P–O–C stretching, the mode at 1173 and 1250 cm^−1^ are specific for the symmetric and asymmetric stretching of the C–O groups, the mode at 1466 cm^−1^ is accompanied by the methylene group from the lipids, the mode at 1739 cm^−1^ represents the stretching vibrations of the ester carbonyl groups (C=O), the mode at 2922 cm^−1^ is specific for the asymmetric stretching vibration in the CH_3_ groups, and the mode at 2853.1 and 3018 cm^−1^ represent the symmetric and asymmetric C–H stretching [52]. Since the FT-IR spectra of the empty (control) liposomes (Figure 4a) showed all characteristic bands of phospholipids, it can be concluded that no chemical reaction occurred during the liposomal preparation. The *P. tenuifolia* L. petal extract FT-IR spectra (Figure S2) display a prominent peak at 1023 cm^−1^ that is indicative of the C–C stretching of the (CH_2_)_n_ group [53], and a mode at 761 cm^−1^ that can be assigned to the carbon skeleton vibration [54]. The band at 1203 cm^−1^ is assigned to the C–O stretching originating from aromatic esters, while the phonon which was observed at 1320 cm^−1^ was associated with the bending vibrations of the CH_3_ and CH_2_ moieties [55], and the band at 1450 cm^−1^ is related to the δ(C–H) deformation. The vibrational mode present at 1606 cm^−1^ in the extract may be assignable to the C=C stretching modes specific for saponins [56]. The band at around 1704 cm^−1^ was attributed to the stretching vibration of the carbonyl group and was present only for hydrolysable tannins [57]. The modes at 2854 and 2924 cm^−1^ are assigned to the symmetric and asymmetric vibrations ν(C–H) of the CH_2_ and CH_3_ aliphatic groups from the alkyl groups, and the broad band peaking at around 3270 cm^−1^ tentatively corresponds to the OH stretching modes [58].

The FT-IR spectra of the liposomes loaded with the *P. tenuifolia* L. petal extract (Figure 4) show a mode that is unique to this petal extract and is located at 1375 cm^−1^. This mode is associated with anthocyanins, which were previously discovered in the chemical composition of the *P. tenuifolia* L. petal extract by Čutović et al. [3]. Anthocyanins are present in the low-intensity band, which is consistent with their origin in the petal extract and suggests that they can be “entrapped” on the liposomal surface. Additionally, the FT-IR spectra of pure petal extract are observed to lack certain modes, such as the bands at 1203 cm^−1^ associated with C–O stretching from aromatic esters in the extract and at 1606 cm^−1^ linked to potential C=C stretching modes indicative of saponin presence. It is possible that the compounds containing these functional groups are now located within the liposomal bilayer and are not detectable in the spectra. According to the literature, when the active compound peaks are obscured, it suggests that the carrier has entirely encapsulated them, which in our instance indicates that the extract compounds were well wrapped during liposome entrapment [34]. In the FT-IR spectra of the petal extract-loaded liposomes, there are also observed modes that belong to the liposome phospholipids, including the modes at 2922, 2856, 1734, 1469, 1251, 1171, 969, 819, and 717 cm^−1^. However, the mode at 576 cm^−1^ assigned to the O–CO–C of the present phospholipids decreased in its intensity, whereas the bond at 918 cm^−1^ corresponds to the phospholipid C–C=O stretching in the FT-IR spectra of the plain liposomes, but has not observed in the spectra of the extract-loaded liposomes. The asymmetric stretching phonon at 1241 cm^−1^ is reportedly affected by the distribution of the encapsulated chemicals both inside and outside of the liposomes [40]. The band is moved from 1241 to 1250 cm^−1^ in the case of the *P. tenuifolia* petal extract-loaded liposomes, showing that the extract components are present in the liposoluble phospholipid tails and on the liposome surface. Additionally, the band linked to the symmetric PO_2_^−^ stretching (1057 cm^−1^) is affected when the encapsulated components are only on the liposomal surface [59]. The fact that the aforementioned band is the same in both the plain and extract-loaded liposomes provides additional proof that *P. tenuifolia* L. petal extract chemicals are present both within and outside the liposomes. The acquired results and conclusions are consistent with the data from the literature, where FT-IR analysis revealed that the plant extract was combined with the membrane interface of the bilayer, as well as the interior of the liposomes [60]. The phonon corresponding to the ν (C–H) ester carbonyl in plain liposomes shifted from 1736 to 1739 cm^−1^ in liposomes loaded with the petal extract. The formation of a hydrogen bond between the carbonyl groups of the phospholipids and the –OH groups found in the extract components caused the modifications in the aforementioned peak [39]. The symmetric and asymmetric C–H stretching in the lipids is represented by the bands at 2857 and 3101 cm^−1^, whereas the asymmetric stretching vibration in the CH_3_ groups is associated with the band at 2922 cm^−1^.

The spectra of the extract-loaded CMC film formulation are shown in Figure 5a. The extract-loaded CMC film displays a heterogeneous peak pattern that ultimately displays the polymer and active extract peaks of the *P. tenuifolia* L. petals. The hydrophilic functional group (O–H) or N–H functional group was identified by the peaks at 3276 cm^−1^ [61]. The cyclic alkene group C=C stretching was represented by the distinctive band at 1584 cm^−1^. The peaks at 1417 and 1319 cm^−1^ were identified as O–H bending typical of carboxylic and alcoholic acids [62]. The confirmation of secondary alcohol (–CH–OH in the cyclic alcohol C–O stretch) was indicated by the band at 1041 cm^−1^. The total findings made it abundantly evident that there is no polymer extract interaction and that *P. tenuifolia* L. petal extract has been successfully incorporated into CMC formulations [63]. Figure 5b represents the CMC films loaded with liposomes composed out of Phospholipon and *P. tenuifolia* L. petal extract. The presence of the bonds at 2928, 2855, and 1597 cm^−1^, which are characteristic for the used petal extract, indicate the presence of the extract indicating that the extract present in the liposomes has been enmeshed into the CMC films. The remaining characteristic peaks pointed out in Figure 5b are also present in the FT-IR spectra of the blank liposomes, indicating their incorporation into the film surface, as well.

### 4.3. Moisture Content

Because *P. tenuifolia* L. petal extracts are sensitive to elevated temperature and there is a threat of bioactive compounds becoming inactive, the films containing the petal extracts contained a slightly higher amount of moisture than the ones in which the extract was already encapsulated before being incorporated into the films [47]. For treatments with liposomes, the measured moisture content was somewhat lower than those containing free *P. tenuifolia* L. petal extracts. This indicated that encapsulation lowers the evaporation of the petal extract during film drying. Films containing liposomes, on the other hand, have a lower water-binding capability. The interactions between the liposomes and the hydrophilic regions of the CMC chains might explain why the film water-binding ability is reduced. The results found in the literature are in agreement with those of our research, pointing out that the addition of plant extract reduces the moisture content of the films [64], as the moisture content of pure CMC films is higher than 95% [26]. The further decrease in the moisture content when liposomes containing *P. tenuifolia* L. petal extracts are mixed into CMC films, on the other hand, might be attributed to the interaction in the film matrix and a reduction in the availability of reactive hydrophilic groups to interact with moisture [65]. 

### 4.4. Stability Study

According to the literature, processes that give a high energy input of agitation, such as the proliposome approach employed in this work, result in the creation of smaller particles than other ways, such as the thin film procedure [33,34]. According to Isailovic et al. [33], the proliposome method uses a small amount of ethanol that can have an effect on the liposome size by lowering the parameter. Ethanol, in particular, alters the net charge of the system, which stabilizes the stearic fluid. The liposomes with petal extracts had a significantly smaller diameter than their empty parallel, in the case where Phosal 75 SA was used as a lipid base (591.53 ± 64.57 nm, Figure 6a). However, in the case of the latter two types of liposomes, the blank ones had a higher value of vesicle size, than the ones containing extracts, which could be due to the fact that they were interlinked according to different interactions than the ones with Phosal 75 SA.

The PDI value is a particle size distribution measurement [66]. The obtained results for the extract-loaded liposomes were lower (~0.615, Figure 6b), whereas for the plain ones, the value was much higher (~0.923, Figure 6). According to Zhao et al. [67], a higher lipid content, may cause a higher PDI value. A PDI value of about 1.0 implies a highly broad size range or the presence of big particles or aggregates, which might contribute to liposome sedimentation [68], thus indicating that the phenomena that occurred in this study, whereby the PDI values decreased with the addition of the petal extract, lead to a high-to-medium homogeneity, making them more stable.

The zeta-potential determined right after the extract-loaded liposomes were prepared ranged from −30.1 to −11.60 mV (Figure 6c). By determining their electrostatic attraction to one another, the physical stability of vesicles can be dramatically affected by the zeta-potential [34]. As a result, the negative and high value of the zeta-potential (as a measure of liposomal stability) established in our case allows for strong electrostatic stabilization of the system, preventing particle aggregation and fusion [34,69]. The behavior of the nanoparticles, their encapsulated contents, and their removal in vivo are also significantly influenced by the surface charge. The anionic nanoparticles have a strong interaction with the reticuloendothelial system cells, scavenging the endothelial cells and blood resident macrophages, whereas the cationic liposomes are quickly cleared from the circulation through a combination of non-specific cellular interactions (adsorption to the anionic surface of the blood vessel walls) and clearance by the reticuloendothelial system specialized cells [70]. The *P. tenuifolia* L. petal extract-loaded liposomes showed a lower zeta-potential (absolute value) compared to the plain liposomal bilayer (ranging from −30.1 to −34.13 mV, Figure 6c). The occurrence that the zeta-potential of extract-loaded liposomes had a lower absolute value that in the case of blank liposomes could be due to the fact that extracts as an additional component of liposomes may undergo oxidative and hydrolytic degradation during the preparation, leading to a shorter shelf life [71]. 

### 4.5. Rheological Characteristics and Physical Properties

Increases in density have the tendency to reduce the thickness of the liposomes, which is advantageous for their application. Additionally, a drop in liposome thickness brought on by an increase in density, has a direct impact on how well the liposome performs because it alters crucial factors, including its opacity, color, and permeability to gases and water [29,72]. The results from Table 1 show that the LPh + petals liposomes had the highest density on the 1st day, with the most significant increase in its value after 60 days of storage; this could be due to the agglomeration of vesicles during storage [73]. The decrease in the density of liposomes leads to a higher fluidity, making them less stable for use in formulations that are intended for longer storage periods [74]. 

Table 1 shows that only for the SA 75 petal-extract loaded liposomes did the storage period of 60 days cause a noticeable decrease in the values of surface tension, reaching the value of 9.23 ± 0.185 mN/m, which is a 58.33% decrease in surface tension. This might be because these formulations utilize a different type of lipid base with a greater percentage of phosphatidylcholine (partially separated from soy lecithin), leaving residues of the surfactant soy lecithin behind [75]. A higher concentration of soy lecithin would result in a lower value for the surface tension of the liposomes because hydrogen bridge formation and other forces involved in molecular adhesion are inhibited by surfactants, which causes the surface tension to decrease with surfactant concentration [76]. Furthermore, it has been discovered that the addition of fatty acids, particularly those with long chains, changes the surface tension readings of liposomes [77]. The smaller changes in the surface tension of the latter two liposomes could be assigned to the fact that they have lower amounts of soy lecithin in their composition than the SA 75 liposomes. 

With a modification in the lipid utilized as the liposome foundation, the viscosity of the extract-loaded liposomes rose exponentially. This was not surprising given that lipids are known to generate very viscous solutions even at low concentrations. Lipids are also known to act as surfactants, as their mixes have a much lower surface tension (72 mN/m at 25 °C) than pure water [78]. Additionally, the liposomes comprising different lipids, as is the case here, are expected not to have the same viscosity due to the different composition of the lipid mixtures, which could lead to a contrast in membrane rigidity, and thus to varying fluidity of liposomes [79]. 

It is thought that the surface tension is related to its molecular weight, and a high interfacial tension will result in the formation of thick layers with poor moisture penetrability, thus leading to a higher stability of liposomes [80]. When the surface tension of the samples decreases, as it has in our study, it can lead to a change in the organoleptic characteristics of the surface, making them less stable [81]. The decrease in viscosity also decreases the stability of the liposomes by leading to increased sedimentation of the particles [82], which could lead to the conclusion that the liposomes are somewhat fairly stable, as the decrease in the values after the 60-day period was small.

### 4.6. Mechanical Properties of Liposome- and Extract-Loaded Films

The addition of *P. tenuifolia* L. petal extract to the CMC films made the films more flexible and deformable due to the molecular interactions that have been detected between the petal extract and the polymer chains present in CMC (Table 2), which has been confirmed via FT-IR analysis outlined above. Interestingly, the inclusion of liposomes had no effect on improving the mechanical characteristics of the CMC-based films. This might be due to various opposing effects. Liposomes, in particular, would be predicted to disrupt the CMC matrix, weakening it.

On the other hand, it has been observed that the inclusion of commercial plasticizers (mixtures of different phospholipids and fats), alone or in conjunction with other types of surfactants, boosted the tensile strength of CMC films [83]. The reason for this was that positively charged phospholipid choline groups might interact with CMC anion groups in order to produce stronger chemical connections, increasing the film’s tensile strength. It is important to note that the film containing liposomes displayed extremely high levels of mechanical parameter variability (standard deviations ranged from 21% to 34%), indicating an inhomogeneous structure. 

The CMC films containing 0.2 g of the *P. teniofolia* L. petal extract had the largest elongation at break (42.75%), which might be attributed to intermolecular interactions between the CMC chains and the extract. Dayarian et al. [84] observed the importance of molecular interactions between film components on material flexibility. The addition of the liposomes into the formulation of the CMC-based films leads to a decrease in the film’s elongation, probably due to the interactions between the choline groups present in phospholipids and CMC anion groups, leading to lower flexibility. 

The mechanical properties of biopolymer films formed from their aqueous solutions depend on many parameters, such as the molecular mass and environment conditions (the thermal conditions, humidity, etc.) [85,86], and they are important as they show how much strain the film can endure, before breaking, which is of high importance for their potential use in the pharmaceutical industry, for the preparation of skin patches. The mechanical properties do not influence the stability of the biopolymer films. 

### 4.7. Biological Activities

#### 4.7.1. Antibacterial Activity of Liposomes

As shown in Table 3, the MIC values of liposomes ranged from 0.5 to 4.0 mg/mL for bacteria. The results indicated that incorporation of the *P. tenuifolia* L. petal extract into the liposomes was more effect against *S. aureus* than the control drug (gentamicin). In the case of the remaining two bacterial strains, *S. lugdunensis* and *P. vulgaris*, extract-loaded liposomes showed lower capability to inhibit their growth than the control drug. 

According to the results, the MIC values of the liposome formulations were lower for Gram-positive bacteria (*S. aureus*). It has been previously reported by Čutović et al. [3] that Gram-positive bacteria are more susceptible to *P. tenuifolia* L. petal extract in comparison to Gram-negative bacteria. The variation in the structural makeup of the cells may be the explanation for this alteration. The high quantities of peptidoglycan in Gram-positive bacterial cell wall allow for substances to penetrate the cells. However, in Gram-negative bacteria, a lipopolysaccharide-based double-layered membrane is connected to the inner membrane, which not only contributes to the antigenicity, toxicity, and virulence of these bacteria, but also functions as a barrier to permeability [87]. The hydrophilic and hydrophobic phenolic compounds, including flavonoids, aromatic esters, and acids that are present in the composition of *P. tenuifolia* L. petal extracts are the basis for the molecular underpinnings of the substance’s antibacterial activity. Because these chemicals are directly attached to the bacterial cell wall, antimicrobial action occurs [88,89]. Besides this, the lipid components of the liposomes interact with the hydrophobic metabolites from the bacteria, thus leading to the suppression of their growth [90]. When the petal extracts are encapsulated into liposomes, micelles are formed, in which the hydrophobic components are kept inside the vesicles, while hydrophilic are in direct contact with bacterial cells, thus exhibiting synergistical antibacterial activity of both the liposomes and the extract. 

#### 4.7.2. Antifungal Activity of Liposomes

The broth microdilution test was used to evaluate the MIC and MFC (mg/mL) of *P. tenuifolia* L. petal extracts integrated into liposomes against various *Candida* strains (Table 4). Phospholipids are suitable carriers for plant extracts because their lipid bilayer structure mimicking cell membranes allows for fusion with fungal membranes, releasing the entrapped extract onto cell membranes or the inner part of the microorganisms. Table 4 shows the MFCs of the petal extracts, blank liposomes, and extract-loaded liposomes against three *Candida* strains. The MFC values ranged from 1.0 to 16.0 mg/mL for blank liposomes, while for the extract-loaded ones, the range was from 0.5 to 4.0 mg/mL. Among the *Candida* species that were used for testing, all three were equally sensitive to the influence of the pure petal extract, whereas in the case of extract-loaded liposomes, *C. krusei* was the fungi most susceptible to their influence, followed by *C. albicans*, making *C. kefyr* the most resistant *Candida* strain. The average MFC for extract-loaded liposomes was about twice that of the pure extract. By the liposomes ability to mimic cell membranes, the interaction of the bioactive compounds, from both the phospholipid mixture and petal extract, with the fungal cells is eased, thus allowing for the antifungal potential [91]. Extracts encapsulated into liposomes have a prolonged period of activity in comparison to the free petal extract, due to the controlled release of the bioactives. 

#### 4.7.3. Antibacterial Activity of Liposome- and Extract-Loaded Films

The film containing pure *P. tenuifolia* L. petal extract seemed to present significant antibacterial activity against *S. aureus* and *S. lugdunensis*, as well as *P. vulgaris*. The films prepared with pure petal extract had an inhibition zone ranging from 13.7 to 30.17 mm depending on the tested bacterial strain and the amount of the extract incorporated into the films, which was significantly higher than in the case of the liposome-loaded films, which had no activity in suppressing the growth of the examined *Staphylococcus* strains. As expected, the incorporation of loaded liposomes into films lowered the antimicrobial capacity of the films.

The inhibition zone of the films made with free *P. tenuifolia* L. petal extract was much greater than that of the films prepared with encapsulated petal extract, as indicated in Table 5. These findings might be ascribed to the encapsulating of biologically active compounds within liposomes, which restricts their release onto the plate surface and resulting in the current compounds having minimal antibacterial activity [92]. Imran [93] had similar findings, since it was discovered that the release of bioactive substances trapped on nanoliposomes is a very drawn-out process.

#### 4.7.4. Antifungal Activity of Liposome- and Extract-Loaded Films

Table 6 shows the diameter of the zone of the growth inhibition of *C. kefyr* and *C. krusei* on the tryptic soy agar (TSA) medium with liposome-loaded films and those containing only pure *P. tenuifolia* L. petal extracts. The zone of inhibition was the highest for the films containing 0.4 g of the petal extract, reaching a value of 35.17 mm against the growth of *C. krusei*. The greater microbial growth in the plates coated with the film that was loaded with liposomes than in those containing the extract-loaded ones can be observed in Table 6. This indicates that they did not have antimicrobial activity, as expected for liposome-loaded films. The incorporation of liposomes did not improve the antifungal potential of films. In this situation, a couple more days of investigation would be required to determine whether there is considerable antibacterial action. These findings might be explained by the active compounds’ liposomal encapsulation, which inhibits their release on the plate surface, as well as the restricted antifungal activity of *P. tenuifolia* L. petal extracts following encapsulation. 

According to Imran’s [93] research, liposome composition is a crucial component to consider when controlling the release of active compounds. In addition, Imran et al. [94] observed that the highest antimicrobial activity corresponded with films that contained both free and encapsulated antimicrobial compounds. This finding shows that a modest quantity of bioactive chemicals must be present in free form at first to prevent microbial growth until bioactives are released from the liposomes.

#### 4.7.5. Antibiofilm Activity of Extract-Loaded and Empty Liposomes

Our previous work [3] has shown a promising ability of the extract of *P. tenuifolia* L. petals collected in Gulenovci to inhibit the formation of *S. lugdunensis* biofilms. The results of this assay are presented in Figure 7, and they point out that the liposomes composed of *P. tenuifolia* petals and Phospholipon present the highest antibiofilm potential. This type of liposome showed the effect of antibiofilm action which is twice higher than in the case of the remaining two types. Thus, it could be assumed that this is due to the synergistic effect of the high polyphenol content of the extract [3] and the lipids present in the Phospholipon. The combined effect of the liposomes made up of Phosal 75 SA, or Phosal 53 MCT and *P. tenuifolia* petal extracts could possibly be due to the more complex interactions between the lipids and the extract, resulting in a longer period needed for the bioactive components to get in contact with the pathogens [93].

### 4.8. Release Study

As shown in Figure 8, polyphenols diffused fast from the extract solution, and the amount of polyphenols in the acceptor compartment peaked after about 200 min. It was to be expected that the release of polyphenols from liposomes would be delayed. After roughly 500 min, the steady state was reached. These findings suggest that liposomes can hold onto polyphenols and be employed for their extended release, which is important for practical use. Concerning the impact of distinct lipids utilized in liposome preparation, it is apparent that for liquid lipids (Phosal 75 SA and Phosal 53 MCT), the presence of cholesterol and/or β-sitosterol from the oils (medium-chain triglycerides or safflower oil) enhances membrane fluidity, resulting in a reduced retention of polyphenols in these liposome forms [95]. Since Phospholipon only consists of a mixture of solid lipids, the membrane in this instance was stiffer, which caused the polyphenols in these liposomes to be released over an extended period. Consequently, ~69% of the total polyphenols (TPs) were liberated from the solution of the extracts after 90 min, and ~38% of TP were released from SA 75 liposomes loaded with petal extract, and about 54% of TPs were released from MCT 53 liposomes with extract compared to 12% in the case of liposomes containing Phospholipon. This discovery is directly tied to the membrane’s fluidity, which is altered in the case of liquid lipids, by the presence of oils.

The polyphenol release from the CMC films is also presented in Figure 8, and it can be seen that their release is much slower than in the case of liposomes, as expected. The maximum amount of polyphenols released after 24 h from the CMC films was ~47%, which is the amount that was released from the solution after only 45 min. This could be due to the fact that an equilibrium is reached in a shorter amount of time, as the film begins to swell, thus closing the pores of the acetate cellulose membrane [26]. When the CMC film was loaded with the Ph liposomes containing the *P. tenuifolia* L. petal extract, the TP release was slightly lower than in the case when the extract was encapsulated only into the film. This type of finding suggests that double encapsulation of extracts (into liposomes and then into films) increases the ability to retain polyphenols. It is expected that this type of formulation retains even more polyphenols, but the lipids from the liposomes tend to react with the polymer chain of CMC, forming micelles, thus leading to the orientation of the hydrosoluble components toward the accepting unit of the Franz diffusion cell. 

Diffusion coefficients and diffusion resistances produced from the liposome bilayer and the film were determined by analyzing data from the release studies. Based on the slope of the linear portion of the curve established by graphing $ln(\frac{{CD}^{0}-{CR}^{0}}{CD-CR})$ vs. time, t, the diffusion coefficients, D, of polyphenols from liposome dispersion were calculated (Figure S3). The findings are shown in Table 7 (column 2). Column 3 of Table 7 presents the results of the calculation of the total diffusion resistance using the following equation: $\ln\left(\frac{C_{D}^{0}-C_{R}^{0}}{C_{D}-C_{R}}\right)=D\beta t$ (where C_D_^0^ stands for the starting concentration of the polyphenols in the donor part of the Francz diffusion cell, C_R_^0^ for the starting concentration of polyphenols in the acceptor part of the Franz cell, C_D_ and C_R_ stand for concentration of the polyphenols in the donor and acceptor part after a period of time, t, and β represents the geometrical component for the standard 20 mL Franz diffusion cell, 2.49 × 10^−4^ m^2^).

Given the decrease in bilayer permeability, it was evident that the various methods used to encapsulate the *P. tenuifolia* L. petal extracts would enhance diffusion resistance. As a result, when a Phospholipon-containing formulation was utilized instead of Phosal 75 SA or 53 MCT lipids for liposome formulation, the bilayer resistance was two times higher. On the other hand, as expected, the highest resistance to polyphenol diffusion through the acetate cellulose membrane was in the case of films containing petal extract-loaded liposomes composed of Phospholipon. This could be due to the interactions between the polymeric chain of CMC and the lipid component of the liposomes, leading them to form micelles, which halt the release of polyphenols into the acceptor section of the diffusion cell [26].

### 4.9. SEM Analysis of Biopolymer Films

The material structures of carboxymethyl cellulose films were examined using SEM, as shown in Figure 9. Via SEM scrutiny of the surface of the petal extract-loaded films, as well as liposome-loaded ones, from Figure 9a,b, it can deduced that the structure of the biopolymer films changed following the addition of liposomes, as they are visible in its structure, while the ones containing the free extract are homogenous. Figure 9b shows some level of flattening in the case of the larger liposomes, but there is no proof that the liposomes ruptured. Figure 9c presents the cross-section of the extract-loaded biofilm, and it can be seen that it is compact, as the film layers are of equal thickness, and the extract is uniformly encapsulated into them. 

## 5. Conclusions

The outcomes of this study demonstrated simple, one-step techniques that could be used to encapsulate the *P. tenuifolia* L. petal extract, rich in a variety of bioactive compounds, into liposomes and biopolymer films. The encapsulation efficacy varied depending on the lipid that was used for the liposome preparation, but was the highest for Phospholipon, which is why this type of petal extract-loaded liposome was chosen to be encapsulated into films. The chemical characterization of all liposomes and films confirmed that the encapsulation process was successful, making the release of the bioactive components of the extract slower. The extract-loaded liposomes containing Phospholipon proved to be the most efficient in inhibiting both bacterial and fungal growth and bacterial biofilm formation. However, when encapsulated into CMC films, neither bacterial nor fungal growth inhibition was achieved, suggesting that insufficient amounts of the bioactive substances were released. 

In conclusion, Phospholipon is a better lipid option for the preparation of liposomes loaded with *P. tenuifolia* L. petal extract than Phosal 75 SA or Phosal 53 MCT. However, the encapsulation of these liposomes into biopolymer films is unfavorable for the release of bioactive substances required for the extract′s skin-beneficial properties. Therefore, further research should be directed toward the development of creams and butters using *P. tenuifolia* L. petal extract-loaded Phospholipon liposomes.

## Figures and Tables

**Table 1:** Density (ρ), surface tension (γ), and viscosity (η) of *Paeonia tenuifolia* L. petal extract-loaded liposomes.

Sample	1st Day			60th Day		
	ρ, g/cm^3^	γ, mN/m	η, mPa∙s	ρ, g/cm^3^	γ, mN/m	η, mPa∙s
L75 SA + petals	1.056 ± 0.02 ^b^*	22.15 ± 0.87 ^a^	130.0 ± 3.11 ^b^	1.132 ± 0.07 ^b^	9.230 ± 0.18 ^c^	121.72 ± 2.15 ^b^
L53 MCT + petals	1.031 ± 0.02 ^c^	22.13 ± 0.93 ^a^	96.97 ± 1.58 ^c^	1.098 ± 0.05 ^c^	18.33 ± 0.07 ^b^	91.57 ± 3.03 ^c^
LPh + petals	1.066 ± 0.02 ^a^	22.76 ± 1.12 ^a^	268.4 ± 2.84 ^a^	1.151 ± 0.08 ^a^	19.57 ± 0.08 ^a^	229.97 ± 4.12 ^a^

* different letters in each column show a statistically significant difference (*p* < 0.05; n = 3, one-way ANOVA, analysis of variance, Duncan’s post hoc test).

**Table 2:** The mechanical characteristics of various carboxymethyl cellulose-based films.

Sample	Film Thickness, mm	Tensile Strength, MPa	Break Force, N	Elongation, %
Film + 0.2 g of extract	0.11 ± 0.02 ^b^	29.25 ± 3.27 ^b^	16.14 ± 5.90 ^a^	42.75 ± 12.67 ^a^
Film + 0.4 g of extract	0.14 ± 0.02 ^ab^	35.72 ± 4.78 ^a^	19.81 ± 3.54 ^a^	37.12 ± 12.92 ^a^
Film + LPh liposome	0.16 ± 0.01 ^a^	14.09 ± 4.76 ^b^	1.64 ± 0.34 ^b^	9.16 ± 3.58 ^c^

Different letters in each column show statistically significant difference (*p* < 0.05; n = 3, one-way ANOVA, analysis of variance, Duncan’s post hoc test).

**Table 3:** Antibacterial activity of empty, and *Paeonia tenuifolia* L. petal extract-loaded liposomes (MIC and MBC, mg/mL).

Sample	Bacteria					
	*Staphylococcus aureus*		*Staphylococcus lugdunensis*		*Proteus vulgaris*	
	MIC	MBC	MIC	MBC	MIC	MBC
L75 SA	2.0	4.0	2.0	4.0	1.0	2.0
L53 MCT	1.0	2.0	2.0	4.0	2.0	4.0
LPh	1.0	2.0	4.0	8.0	2.0	4.0
L75 SA + petals	1.0	2.0	1.0	2.0	0.5	1.0
L53 MCT + petals	0.5	1.0	1.0	2.0	1.0	2.0
LPh + petals	0.5	1.0	1.0	2.0	0.5	1.0
Petal extract	0.5	1.0	0.5	1.0	0.5	1.0
Gentamicin, control	1.33	2.66	0.008	0.016	0.066	0.133

Minimal inhibitory concentration (MIC); minimal bacterial concentration (MBC). The study was repeated three times, and the highest value obtained was used as the MIC and MBC (“stricter criteria” rule was used, as is usual in antimicrobial tests).

**Table 4:** Anticandidal activity of empty and *Paeonia tenuifolia* L. petal extract-loaded liposomes (MIC and MFC, mg/mL).

Sample	Fungi					
	*Candida albicans*		*Candida kefyr*		*Candida krusei*	
	MIC	MFC	MIC	MFC	MIC	MFC
L75 SA	4.0	8.0	4.0	8.0	4.0	8.0
L53 MCT	4.0	8.0	8.0	16.0	4.0	8.0
LPh	4.0	8.0	8.0	16.0	0.5	1.0
L75 SA + petals	2.0	4.0	2.0	4.0	2.0	4.0
L53 MCT + petals	2.0	4.0	2.0	4.0	1.0	2.0
LPh + petals	2.0	4.0	2.0	4.0	0.25	0.5
Petal extract	0.5	1.0	0.5	1.0	0.5	1.0
Ketoconazole, Control	0.05	0.1	0.05	0.1	0.05	0.1

Minimal inhibitory concentration (MIC); minimal fungicidal concentration (MFC). The analysis was performed in triplicates, and the highest value obtained was taken as the MIC and MFC (“stricter criteria” rule was applied, common in antimicrobial assays).

**Table 5:** Diameters of inhibition zones in the disc diffusion assay for bacterial strains.

Bacteria	Sample	Zone of Inhibition, mm
*Staphylococcus aureus*	Liposome-Loaded Film	/
	Extract-Loaded Film (0.2 g)	15.34 ± 4.77
	Extract-Loaded Film (0.2 g)	27.83 ± 9.13
	Streptomycin	26.00 ± 1.21
*Staphylococcus lugdunensis*	Liposome-Loaded Film	/
	Extract-Loaded Film (0.2 g)	9.34 ± 1.67
	Extract-Loaded Film (0.4 g)	23.00 ± 4.46
	Streptomycin	27.24 ± 2.44
*Proteus vulgaris*	Liposome-Loaded Film	7.50 ± 1.10
	Extract-Loaded Film (0.2 g)	13.66 ± 5.32
	Extract-Loaded Film (0.4 g)	30.16 ± 13.19
	Streptomycin	27.33 ± 4.53

**Table 6:** Diameters of inhibition zones in the disc diffusion assay for *Candida* strains.

Bacteria	Sample	Zone of Inhibition, mm
*Candida kefyr*	Liposome-Loaded Film	/
	Extract-Loaded Film (0.2 g)	15.16 ± 3.70
	Extract-Loaded Film (0.4 g)	35.16 ± 11.22
	Ketoconazole	25.42 ± 1.22
*Candida krusei*	Liposome-Loaded Film	/
	Extract-Loaded Film (0.2 g)	10.33 ± 1.25
	Extract-Loaded Film (0.4 g)	20.83 ± 5.10
	Ketoconazole	27.48 ± 3.34

**Table 7:** Diffusion coefficients and diffusion resistance of *Paeonia tenuifolia* L. extract, extract-loaded liposomes, and extract-loaded films. D—diffusion coefficient; R—total mass transfer resistance.

Sample	*D*, m^2^/s	*R*, s/m
Extract	2.88 × 10^−8^	* 141,195.8
L75 SA + petals	1.78 × 10^−8^	228,779.7
L53 MCT + petals	1.49 × 10^−8^	272,894.2
LPh + petals	8.50 × 10^−9^	479,176.4
Film + e	1.91 × 10^−8^	5746.154
Film + LPh + petals	1.53 × 10^−8^	8518.421

* Diffusion resistance stemming from the aceto-cellulose membrane.

## Data Availability

Data is contained within the article and Supplementary Materials.

## Supplementary Materials

The following supporting information can be downloaded at: https://www.mdpi.com/article/10.3390/pharmaceutics15122742/s1, Figure S1: Moisture content of the extract- and liposome-loaded films; Figure S2: FTIR spectra of the *Paeonia tenuifolia* L. petal extract Figure S3: Dimensionless plot of the total polyphenol concentration from the *Paeonia tenuifolia* L. petal extract, extract-loaded liposomes, extract-loaded films, and liposome-loaded films versus time for release curves.
