# Supplementary material for: Liposomal and Liposomes-Film Systems as Carriers for Bioactives from Paeonia tenuifolia L. Petals: Physicochemical Characterization and Biological Potential
Source: Pharmaceutics. 2023 Dec 7;15(12):2742. doi: 10.3390/pharmaceutics15122742 (PMC10747293; doi:10.3390/pharmaceutics15122742)
Supplement: Supplementary file 1 [file pharmaceutics-15-02742-s001.zip › Figure S1.pdf]

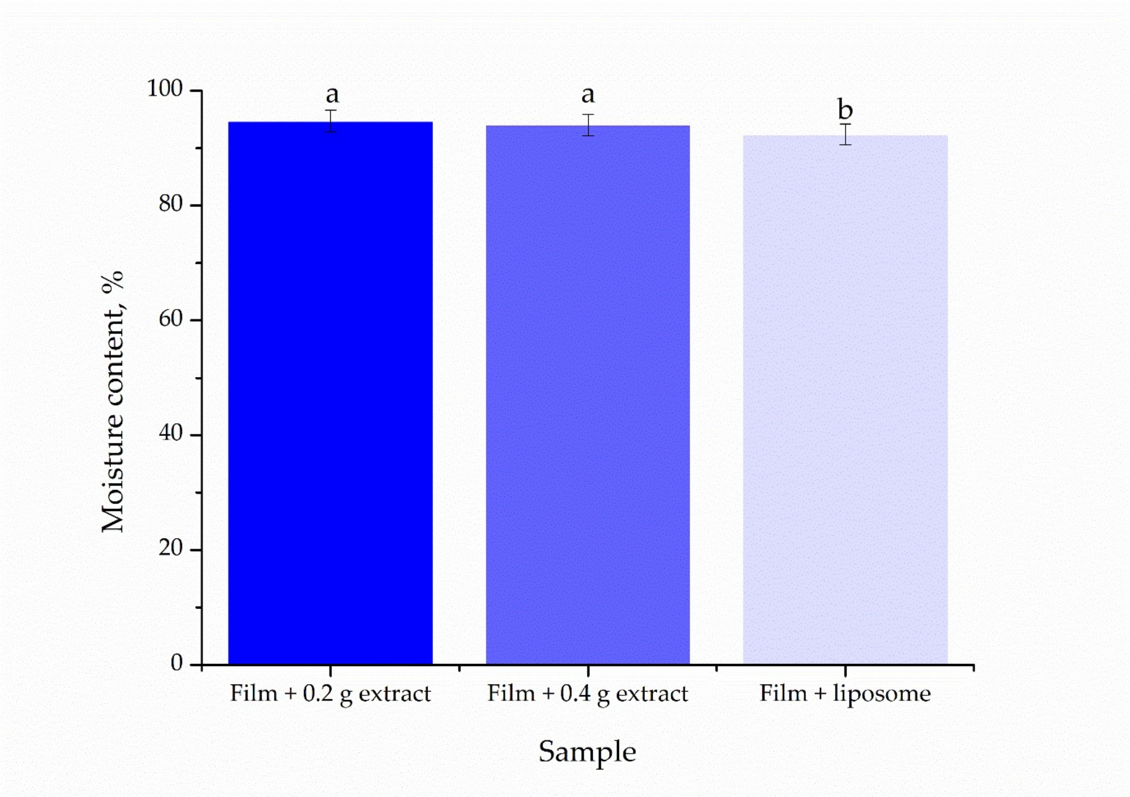

Figure S1 Moisture content of the Paeonia tenuifolia L. extract - and liposome - loaded films; different letters show statistically significant difference ($p < 0.05$; $n = 3$, one-way ANOVA, analysis of variance, Duncan's post hoc test)
