# Supplementary material for: Liposomal and Liposomes-Film Systems as Carriers for Bioactives from Paeonia tenuifolia L. Petals: Physicochemical Characterization and Biological Potential
Source: Pharmaceutics. 2023 Dec 7;15(12):2742. doi: 10.3390/pharmaceutics15122742 (PMC10747293; doi:10.3390/pharmaceutics15122742)
Supplement: Supplementary file 1 [file pharmaceutics-15-02742-s001.zip › Figure S3.pdf]

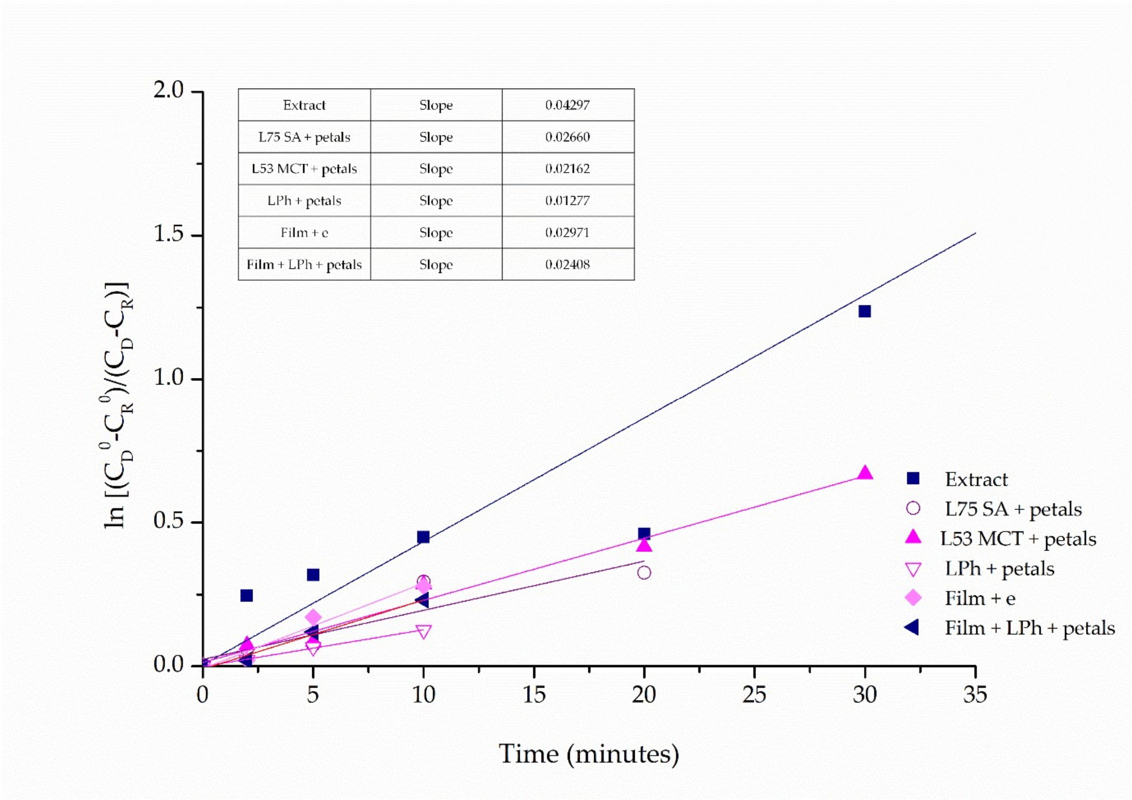

Figure S3 Dimensionless plot of total polyphenol concentration from Paeonia tenuifolia L. petal extract, extract-loaded liposomes, extract-loaded films, as well as liposome-loaded films, versus time for the release curves. (where C_D^0 stands for the starting concentration of the polyphenols in the donor part of the Francz diffusion cell, C_R^0 for the starting concentration of polyphenols in the acceptor part of the Franz cell, the C_D and C_R stand for concentration of the polyphenols in the donor and acceptor part after a period of time, t; L stands for liposome, Ph for Phospholipon, e for extract)
