# Supplementary figures and images for: Liposomal and Liposomes-Film Systems as Carriers for Bioactives from Paeonia tenuifolia L. Petals: Physicochemical Characterization and Biological Potential
Source: Pharmaceutics. 2023 Dec 7;15(12):2742. doi: 10.3390/pharmaceutics15122742 (PMC10747293; doi:10.3390/pharmaceutics15122742)

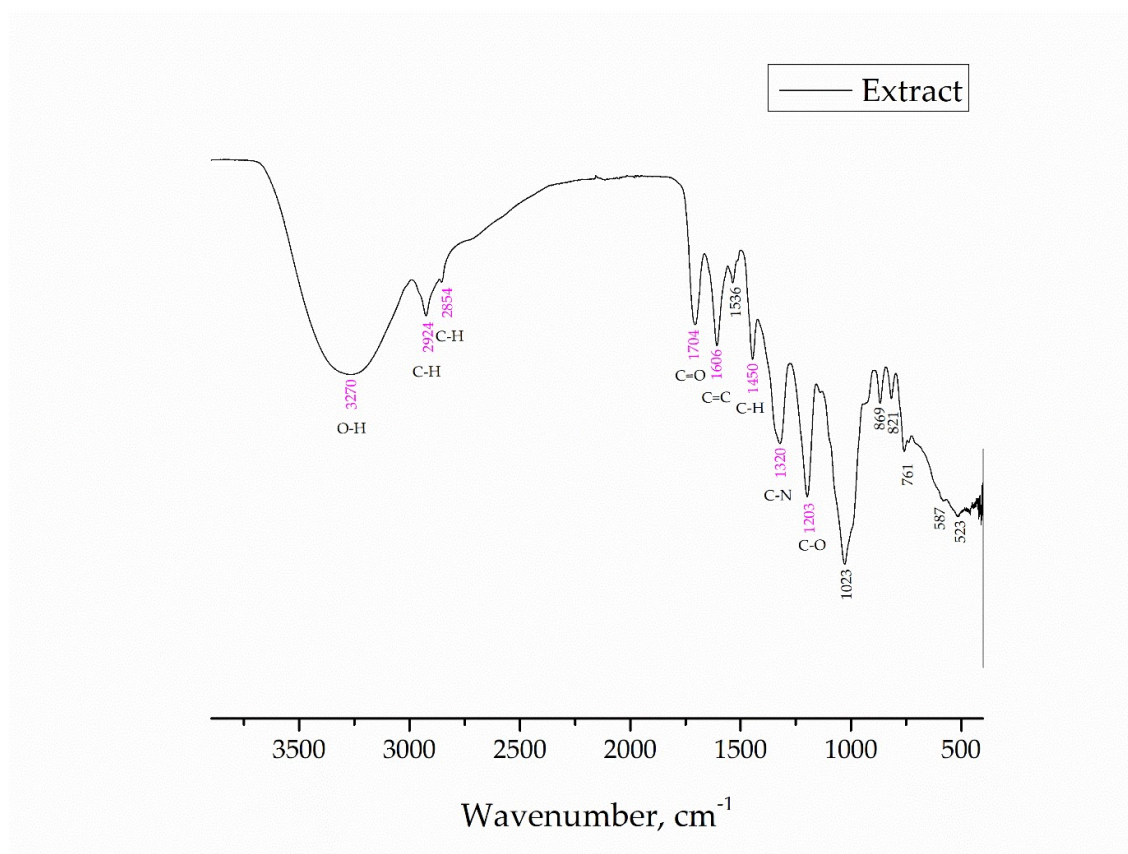

Figure S2 FTIR spectra of *Paeonia tenuifolia* L. petal extract

Supplement: Supplementary file 1 [file pharmaceutics-15-02742-s001.zip › Figure S2.pdf]
